# Acknowledgement to Reviewers of *Micromachines* in 2018

**DOI:** 10.3390/mi10010043

**Published:** 2019-01-09

**Authors:** Micromachines Editorial Office

**Affiliations:** MDPI, St. Alban-Anlage 66, 4052 Basel, Switzerland

Rigorous peer-review is the corner-stone of high-quality academic publishing. The editorial team greatly appreciates the reviewers who contributed their knowledge and expertise to the journal’s editorial process over the past 12 months. In 2018, a total of 673 papers were published in the journal, with a median time to first decision of 15 days and a median time to publication of 38 days. The editors would like to express their sincere gratitude to the following reviewers for their cooperation and dedication in 2018:
Aaen, Peter H.Lin, YangAas, MehdiLin, Chi-YingAbad, Manuel DavidLinß, SebastianAbate, YohannesLinzon, YoavAbdollahzadeh Jamalabadi, M. Y.Liou, MichelleAbdul Samad, YarjanLiparoti, SaraAbdulrahim, MujahidLipfert, JanAbelmann, LeonLiu, YuxinAbhyankar, VinayLiu, QingkunAbidi, KhalidLiu, YazhaoAbolfazl, ZahediLiu, Cheng-HsienAbolhasani, MiladLiu, Han YinAbou-Saleh, Radwa H.Liu, JunAbramovich, HaimLiu, RanAdamu-Lema, FikruLiu, RenAghababaei, RaminLiu, JindongAguilella-Arzo, MarcelLiu, TianyuAhmed, RiazLiu, YangAhmmed, K.M. TanvirLiu, YingAhsan, Md. ShamimLiu, YuAi, YeLivermore, CarolAkafuah, Nelson K.Lo, Yu-HwaAkram, Muhammad NadeemLópez De Lacalle, Luis N.Al-Ameri, TalibLópez Villanueva, Juan AntonioAlessio, VernaLopez-Martinez, Maria JAlfano, BrigidaLu, NanshuAlgorri, José FranciscoLu, MengAli, Dr. Md. AzaharLu, XiyuanAlirezaee, ShahpourLu, YipengAlsaleem, FadiLu, XinyuÁlvarez, MarLucarini, GioiaAmarie, DragosLucchetta, Daniele EugenioAmit, IddoLucibello, AndreaAn, SeongpilŁuczak, SergiuszAn, SangminLudwig, KipAnders, JensLuo, YanhuaAngotzi, Gian NicolaLuo, QuantianAnsari, AzadehLuo, JerryAnsari, FarhanLuo, JianAnthony, CarlLuo, XiaolongAral, MustafaLuo, ShanAramesh, MortezaLutey, Adrian H.A.Arefin, Md. ShamsulLvova, LarisaAriga, KatsuhikoMa, JiajuArnold, WalterMa, JiArscott, SteveMaccaferri, NicolòAryasomayajula, AdityaMaceiczyk, RichardAsadnia, MohsenMachida, KatsuyukiAsokanthan, Samuel F.Maeda, YusakuAssaf, TareqMaeng, JiminAttar, HooyarMagdanz, VeronikaAu, Sam H.Mahshid, SaraAudette, MichelMakeyev, OleksandrAvci, EbubekirMaletinsky, PatrickAw, KeanMalgaretti, PaoloAzar, MassoodMalinauskas, MangirdasAzimi, SoheilMalka, DrorAzizi, EbrahimMallick, DhimanBae, HyungdaeMalm, B. GunnarBaek, DonghyunMalviya, Kirtiman DeoBagdziunas, GintautasMandal, SoumyajitBagolini, AlviseManrique, David ZsoltBai, JingMaoz, Ben M.Balasingam, Suresh KannanMarafona, José DuarteBanerjee, Ashis GopalMarasso, Simone LuigiBannock, James H.Marcos, MarcosBao, YinyinMariani, StefanoBarbato, MarcoMarino, AttilioBarbato, Felicia Carla TizianaMarkopoulos, Angelos P.Barbe, Jean-charlesMarkov, DmitryBarberi, RiccardoMarques, CarlosBardweel, HamzehMartel-Foley, JosephBarfidokht, AbbasMartin, AndréBarletta, DiegoMartinez, Ramses V.Baron, ThomasMasoero, MarcoBarralon, PierreMastropaolo, EnricoBarreto, ArmandoMaterer, Nicholas F.Barshtein, GregoryMatko, VojkoBartlett, Michael D.Matsuhisa, NaojiBartsch, HeikeMatsukawa, YoshitakaBasha, MohamedMatsuo, ShigekiBasit, Munshi MahbubulMautner, AndreasBasu, AnirbanMavridis, IoannisBatra, A. K.Mazomenos, EvangelosBaù, MarcoMcHugh, KevinBauer, RalfMehdipour, ImanBeheim, Glenn M.Mehrabadi, Mohammad AllahdadiBeilliard, YannMeister, JörgBeker, LeventMejia, IsraelBeleggia, MarcoMelo, Luís F.Belfiore, Nicola PioMemoli, GianlucaBella, FedericoMeneghini, MatteoBellantone, VincenzoMeng, FanbenBelo, Joao HortaMeng, EllisBen, YueyangMerazzo, Karla J.Benedic, FabienMercer, JohnBenseman, Timothy M.Meredith, NathanBerg-Sorensen, KirstineMeyer, FlorentBernard, YvesMiah, Abdul HalimBernini, RomeoMichael, AronBertocci, FrancescoMichalik, JanBertsch, ArnaudMiled, AmineBesagni, GiorgioMileti, GaetanoBesozzi, EdoardoMiluski, PiotrBhadra, SharmisthaMinakshi, ManickamBhakhri, VineetMiranda, JoaoBhalla, NikhilMiranda, João M.Bhaskaran, MadhuMirea, TeonaBhattacharjya, DhrubajyotiMirzavand, RashidBhattacharya, SriparnaMishra, Rupesh K.Bianucci, PabloMishra, AashwinBieniek, TomaszMistura, GiampaoloBittner, AchimMitchell-Smith, JonathonBjorninen, ToniMitra, SrinjoyBlack, Bryan JamesMiura, TakashiBlanche, Pierre-alexandreMizoshiri, MizueBleichner, MartinModica, FrancescoBobinger, MarcoMoghimi, MohammadBoek, EdoMohamadi, RezaBogdanowicz, RobertMohammad, MahdaviBoland, Jessica L.Mohd-Yasin, FaisalBorges Sebastião, IsraelMohsen, Marani BarzaniBort, Juan M. AndrésMondal, KunalBose, SaurabhMontessori, AndreaBota, SebastiaMontgomery, Erwin B.Boudaden, JamilaMontoliu Colas, RaulBoudot, RodolpheMoon, Kee S.Bourouina, TarikMorales-Guio, Carlos G.Boussommier-Calleja, AlexandraMori, NobuyaBoutami, SalimMorizio, JamesBoyd, AnthonyMoschou, DespinaBragheri, FrancescaMossi, Karla M.Bramesfeld, GoetzMouza, Aikaterini A.Bratsun, DmitryMu, XuanBrevik, IverMulholland, AnthonyBrisk, PhilipMulla, YusufBrouzes, EricMüller, GerhardBrunet, PhilippeMulloni, VivianaBrunton, EmmaMulvana, HelenBucci, DavideMunteanu, Florentina-DanielaBuj, IreneMuroyama, MasanoriBurugupally, Sindhu PreethamMuszyński, TomaszCabot, Joan MarcNabavi, AliCajal, CarlosNadolny, KrzysztofCakmakyapan, SemihNag, ManojCalabrese, LuigiNagai, MoetoCalaza, CarlosNagatani, NaokiCaliano, GiosueNagayama, TomonoriCalvo, RoqueNaha, Pratap C.Camarchia, VittorioNajarian, SiamakCamarillo, DavidNakano, MichihikoCamboni, DomenicoNam, YoonkeyCaminero, M.A.Nama, NiteshCampos, ManuelNarakathu, Binu BabyCanfield, BrianNasiriavanaki, MohammadrezaCano, Juan LuisNassiopoulou, AndroulaCanpolat, CetinNawaz, SarfrazCao, HuayuNazari Nejad, SamanCaporali, StefanoNelson, Alexander H.Cardenas, JaimeNeri, GiovanniCarey, BenjaminNewcomb, RobertCarou, DiegoNg, AlphonsusCarugo, DarioNguyen, Thanh D.Casas Bedoya, AlvaroNguyen, Duc ThanhCaselli, FedericaNguyen, Tien DatCassidy, John F.Nguyen, Tu (Ryan)Castellanos-Ramos, JuliánNi, GuangxinCataldi, PietroNian, QiongCatarino, SusanaNicolay, PascalCavicchi, KevinNieto, DanielCazzanelli, MassimoNightingale, AdrianCegla, FredericNiklaus, FrankCellini, FilippoNikolka, MarkCeylan, HakanNikumb, SuwasChaisakul, PapichayaNilghaz, AzadehChakraborty, MonojitNirichan, Sanoj RejinoldChalmers, Jeffrey J.Niu, RanChan, PakNivedita, NiveditaChan, King YukNiwano, MichioChan, Yung-KuanNomura, Ken-ichiChandrahalim, HengkyNordin, Gregory P.Chang, BoNose, ToshiakiChang, Yoon-SeokNosrati, RezaChang, Chih-WeiNovak, RichardChang, LingqianNunes, JanineChang, WenlongO’Mahony, ConorChannon, RobertObata, KotaroCharkhkar, HamidObikawa, ToshiyukiCharlton, PeterOchowiak, MarekCharmet, JeromeOgawa, ShinpeiChaspari, TheodoraOh, Min-cheolChaudhary, VarunOhlckers, PerChavoshi, Saeed ZareOk, Jong GChen, ChihchenOliveira, SaraChen, JinjuOlson, Elizabeth S.Chen, Chia-HungO'mullane, AnthonyChen, XingOrsini, AndreaChen, Chi-FengOrtega-Casanova, JoaquínChen, Tao-HsingOrtolani, MicheleChen, Shao-WenOstadhassan, MehdiChen, QiushuOta, HirokiChen, Chia-YuanOxley, ThomasChen, JunPachauri, VivekChen, YuanliuPadmanabhan, JagannathChen, MohanPagliara, StefanoChen, RuiminPagonis, Dimitrios – NikolaosChen, DaiqinPaiè, PetraChen, ChunhuiPak, On ShunChen, HepingPalchoudhury, SoubantikaChen, NanguangPalumbo, FabioChen, ZhenghuaPan, ZhiliangChen, PengyuPandey, SantoshChen, YunhanPandraud, GregoryCheng, ChengPani, DaniloCheng, GuangmingPantano, MariaCheng, ZhenzhouPantoli, LeonardoCheng, ZengguangPapadakis, ChristineCheng, Chiang-hoPark, ChoongbaeChesneau, XavierPark, Yong IlChiang, Cheng-TaPark, Byung-WookChiang, Chia-ChinPark, SungsuChien, Jun-ChauPark, Chun GwonChin, Jit KaiPark, Jang-UngChinappi, MauroPark, Hong-GyuChiolerio, AlessandroPark, Je-KyunCho, Young-RaePark, JunyongCho, HansangPark, Jung-DongCho, ChungyeonPark, JoontaekChoi, Seung-BokPark, JonghyunChoi, WooSeokParker, Stewart F.Choi, JungilPassilly, NicolasChoi, JungwookPatel, SaurinChoi, Hyo-JickPatelli, AlessandroChoi, HeekyuPatterson, JenniferChoi, Kyung HyunPattini, FrancescoChoi, SungyoungPauliukaite, RasaChow, Hwang-CherngPawlak, MichałChristoffer, AbrahamssonPayam, Amir FarrokhChua, BeeleePedersen, Henrik (USA)Chuang, Cheng-HsinPedersen, Henrik (Sweden)Chung, Ying-ChienPeijnenburg, Willie J. G. M.Churchill, HughPeiner, ErwinCimalla, VolkerPekarovicova, AlexandraCinquemani, SimonePellet, ClaudeClement, YuenPeng, ZhiliClimente-Alarcon, VicentePeng, GuimingCogan, StuartPennathur, SumitaCohen, EstellePennazza, GiorgioCoimbatore Balram, KrishnaPereira Neves, HerculesCollin, EddyPeres, António M.Collins, David J.Pérez-Juste, IgnacioComstock, MatthewPerpiña, XavierConinck, Joel DePerrault, CecileConnelly, DanielPersano, AnnaConstantoudis, VassiliosPetruska, AndrewCooke, MichaelPettersson, AndersCopic, DavorPeyman, Sally A.Coppola, SaraPezzinga, GiuseppeCordill, MegalPfattner, RaphaelCortese, BarbaraPfohl, ThomasCoskun, BulutPham, Duc TruongCOURTIAL, Edwin-JofreyPham, PhuongCoy, EmersonPhan, Hoang-PhuongCozzoli, P. DavidePhan, Manh-HuongCrane, NathanPicollo, FedericoCroce, Anna CletaPiedade, Ana PaulaCroissant, JonasPieri, FrancescoCroizet, CédricPinho, DianaCroquette, VincentPinho, LuísCrupi, GiovanniPinteala, MarianaCui, BoPinto, Artur M.Culebras, MarioPirozzi, SalvatoreCulha, UtkuPitto-Barry, AnaïsD’Alonzo, MarcoPlochberger, BirgitDa Silva, Jaime Pedro OliveiraPodder, PranayDagdeviren, CananPoenar, DanielDai, JingPoletkin, KirillDalla Costa, GiuseppePolimeni, AntonioD'andrea, CristianoPongrácz, AnitaD'Andreagiovanni, FabioPoot, MennoDas, PradipPope, TomDash, BirajaPrakash, ShauryaDavaji, BenyaminPrefasi, EnriqueDawson, JeremyPrintz, Adam D.De Aza, Piedad N.Priye, AashishDe Boer, GregProietti, EmanuelaDe Dood, Michiel J.Psychogiou, DimitraDe Gasperis, GiovanniPu, Suan HuiDe La Fuente, Germán FranciscoPuchades, IvanDe Marco, CarmelaPullano, Salvatore AndreaDe Munari, IlariaQamar, AfzaalDe Nijs, BartQasaimeh, Mohammad A.Dean, RobertQian, ZhenyunDefay, EmmanuelQin, YihengDegennaro, LeonardoQin, HantangDehaeck, SamQiu, ZhenDelfs, Jens-OlafQiu, HaoDelivopoulos, EvangelosQiu, YinghuaDellar, Paul J.Qiu, TianDell'Olio, FrancescoQu, HongweiDelnavaz, AidinQuagliata, LucaDeMiguel-Ramos, MarioQue, LongD'Emilia, GiulioRabinovici, RaulDemirci, IbrahimRadhakrishna, UjwalDeng, Nan-NanRaducanu, Bogdan C.Deshpande, S.R.Raeis Hosseini, NiloufarDestgeer, GhulamRaffo, AntonioDevasahayam, SheilaRaj, SaurabhDi Bartolomeo, AntonioRajaraman, SwaminathanDi Benedetto, LuigiRajnicek, AnnDi Caprio, GiuseppeRamanavicius, ArunasDi Schino, AndreaRamini, AbdallahDi Sieno, LauraRamos, DanielDianat, PouyaRanga, AdrianDiban, NazelyRao, SmithaDickert, Franz L.Rapp, Bastian E.Didar, TohidRavasio, ChiaraDiller, EricReboud, JulienDimaki, MariaRegev, MichaelDing, Xiaoyun (Sean)Reid, Russell C.Ding, JunjunReig, CàndidDing, ZhaoyangReigh, Shang YikDing, WenduReineck, PhilippDinh, ToanReinert, WolfgangDixit, Chandra K.Reinhard, FriedemannDo, Thanh NhoRen, HaoDohi, TetsujiRen, YukunDombek, GrzegorzRen, YongDong, JunliangRenevey, PhilippeDong, MingdongRetterer, Scott T.Dong, DaoyiReyes-Garcés, NathalyDrese, Klaus StefanReyssat, EtienneDruffel, ThadRezai, PouyaDryden, Michael DMRezk, AmgadDu, E (Sarah)Rhallabi, AhmedDuan, XiyuRhee, HuinamDubuc, DavidRhi, Seok-hoDuchamp, MartialRho, JunsukDucobu, FrançoisRiha, Shannon C.Duffy, SimonRimoldi, LucaD'Urso, GianlucaRistic, MichaelDusny, ChristianRivadeneyra, AlmudenaDziedzic, AndrzejRiveros, RaulEberle, PatricRizal, ConradEbralidze, Iraklii I.Robinson, Jacob T.Eddington, DavidRocha, Luís A.Eder, StefanRodger, Damien C.Edirisinghe, MohanRodriguez, NoelEftekhar Azam, SaeedRohac, JanEhrmann, AndreaRokosz, KrzysztofElbuken, CaglarRomano, LuciaElg, DanielRosa, ViniciusEl-Heliebi, AminRosengarten, GaryElisa, KallioniemiRosenkranz, AndreasEllett, FelixRosenzweig, DerekElmazria, OmarRossi, MassimilianoElsayed, MohannadRostami, BehnamElshimali, Yahya I.Rothbauer, MarioEmadi, ArezooRout, BhimsenEnguita, José MaríaRufer, LiborEnteria, Napoleon A.Ruffato, GianlucaEral, H. BurakRuffino, FrancescoErdmann, AndreasRughoobur, GirishEreifej, EvonRurali, RiccardoErtl, PeterRussell, ChristopherEsashi, MasayoshiRusu, CristinaEscobedo, CarlosRuther, PatrickEscobedo-Lucea, CarmenRydosz, ArturEtcheverry, SebastianRyu, HyunryulEtienne, DagueSabater, CarlosFacchini, FrancescoSabater-Navarro, José M.Falcucci, GiacomoSabhachandani, PoojaFallon, JamesSadowski, ŁukaszFan, LinranSaeidi, KamranFang, HuiSaez, IgnacioFarokhi, HamedŠafarič, RikoFassi, IreneSagués, FrancescFeiertag, GregorSaharan, LalitaFekete, ZoltánSahoo, DeepakFerhan, Abdul RahimSahu, DebashishFernandez Rivas, DavidSaito, AkiraFernandez-Prieto, ArmandoSaito, ShinichiFerrandiz Bou, SantiagoSaito, KenFerrari, MicheleSajid, MuhammadFerraro, DavideSakata, ShuzoFerraro, PietroSakharova, NataliyaFilippeschi, AlessandroSakudo, AkikazuFinkler, AmitSalek, Mohammad MehdiFinney, Eric E.Salem, BassemFirebaugh, Samara L.Saltaren, RoqueFlowers, GeorgeSaluk-Bijak, JoannaFodor, Petru S.Samiei, EhsanFolch, AlbertSankaran, Kamatchi JothiramalingamFoudeh, AmirSantecchia, EleonoraFoulds, IanSantiranjan Shannigrahi, SantiranjanFournel, FrankSanyal, ArindamFournelle, MarcSardina, GaetanoFrancés-Soriano, LauraSardini, EmilioFrense, DieterSaremi, MehdiFriedt, Jean MichelSarkar, KausikFriedt, Jean-MichelSato, KimiyasuFrisch, BenjaminSaxena, TarunFromen, CathySchenk, HaraldFroriep, Ulrich PSchintke, SilviaFukuda, TakeshiSchirhagl, RomanaFunari, RiccardoSchmidt, Oliver G.Funfschillin, DenisSchmitt, KatrinFung, Wai-KeungSchmitt, MichaelFuso, FrancescoSchneckenburger, HerbertGaczynska, MariaSchneider, MichaelGai, YaSchneider, Ben H.Gaitas, AngeloScholten, KeeGalleani, LorenzoScholz, FerdinandGanesana, MallikarjunaraoSchulz, Sebastian AndreasGanser, ChristianSchwaminger, SebastianGao, JieScott, Amy M.Gao, NanSebastian, VictorGao, YujiSedlmeir, FlorianGao, XuanSegreto, TizianaGao, WeiboSeker, ErkinGarbovskiy, YuriySEKINE, TomohitoGarcía, NuriaSelyanchyn, RomanGarcía-Miquel, HectorSemaltianos, Nikolaos G.García-Sánchez, PabloSemprebon, CiroGargiulo, Gaetano DSeo, JongmoGargiulo, GaetanoSeo, JihoonGebicki, JacekSeok, Tae JoonGębicki, JacekSepúlveda, NelsonGennari, OriellaSerpooshan, VahidGentile, VittorioSerra, Christophe A.Georgiou, Emmanuel P.Serrano-Aroca, ÁngelGerber, DoronSerry, MohamedGerdon, ArenSethian, James A.Ghafar-Zadeh, EbrahimSethu, PalaniappanGhayesh, MergenSetti, DineshGiakos, GeorgeSeymour, JohnGiani, AlainShah, PratikkumarGiannakoudakis, DimitriosShahab, ShimaGiannetti, AmbraShalaby, MohamedGibbs, JohnShan, XuechuanGielen, FabriceShang, LuoranGiménez-Gómez, PabloShao, LeiGimsa, JanSharma, SwatiGiorgio, IvanSharma, DeeptiGiubileo, FilippoShaw, KirstyGizdavic-Nikolaidis, MarijaShayesteh Moghaddam, NargesGlowacz, AdamShaygan, MehrdadGoel, SauravShemelya, CoreyGogneau, NoelleShen, XilingGokhale, Vikrant JayantSherrott, Michelle C.Golub, Victor V.Sherwood, Joseph M.Gomez, AlessandroShi, HongliangGómez Salinas, DídacShields, WyattGong, HuaShields IV, C. WyattGonzález, IcíarShih, SteveGonzález, María UjuéShih, Wen-PinGonzález-Romero, ElisaShikida, MitsuhiroGoossen, KeithShimada, KeitaGopalan, Sai AnandShimazoe, KenjiGordillo, Jose ManuelShimizu, KazuoGrattieri, MatteoShin, SangwooGray, Michael D.Shoffstall, AndrewGreene, NathanielShrirao, Anil B.Greener, JesseShyu, Jin-CherngGriffini, GianmarcoSidorenko, AlexanderGrigsby, Christopher L.Sieber, IngoGrijalvo, SantiagoSier, Cornelis F.M.Grondin, FrancoisSiller, Hector R.Gross, Andrew JamesSilva, Francisco J. G.Grossi, MarcoSimmchen, JulianeGrünberger, AlexanderSimpson, M. CatherGspann, ThuridSingh, AbhalaxmiGu, XiaodanSirutkaitis, ValdasGu, WentingSmulko, JanuszGuan, WeihuaSo, HongyunGuaraldo, Thais TassoSoejima, TetsuroGuder, FiratSohgawa, MasayukiGuénolé, JulienSohn, Jung WooGuijt, Rosanne M.Soin, NavneetGuiney, Linda M.Soltani, AliGuix, MariaSomà, AurelioGumuscu, BurcuSon, DongheeGunkel, IljaSon, Seung UkGuo, PeijunSong, Young MinGuo, ZhanhuSong, XuGuo, QingboSong, Yong-Ak (Rafael)Gutruf, PhilippSong, JingfengGuzman, MarceloSong, JuhaHaddadi, AbbasSongmene, VictorHADDAG, BadisSonker, MukulHaesler, JacquesSorgato, MarcoHafiz, Md Abdullah AlSoria, SilviaHaghshenas, MeysamSortino, MarcoHaidar, RiadSosnowski, Tomasz RobertHakem, NadirSpeghini, AdolfoHalldórsson, SkarphéðinnSpencer, NicholasHamon, MorganSpiers, AdamHan, Yen-LinStassi, StefanoHane, KazuhiroStavrakis, StavrosHao, GuangboStavroulakis, Georgios E.Hao, NanjingSteenson, David PaulHarnois, MaximeSteiner, HaraldHasanov, NamigSterken, TomHashim, HairulazwanStiharu, IonHasni, HasseneStine, Keith J.Hassan, Sammer-ulStolov, Andrei A.Hassanin, HanyStratakis, EmmanuelHattar, KhalidStringer, Jonathan EdwardHayakawa, TohruStrohm, EricHe, MeiStruk, PrzemyslawHe, YongSu, ZhijuanHeepe, LarsSubramani, ThiyaguHegemann, DirkSuess, Ryan J.Heise, BettinaSullivan, JohnHejazian, MajidSun, KeyeHellström, Per-ErikSun, ChenHemsel, TobiasSun, XiankaiHerkommer, AloisSun, Yung-ShinHernando, JorgeSun, DaliHerrera-May, AgustínSun, JianHerrero, RebecaSun, ShijingHerth, EtienneSun, Kien WenHesketh, PeterSun, MengHida, HirotakaSun, HongyuHidrovo, Carlos H.Sung, JaeyongHiggins, SimonSunter, Jack D.Hinchet, RonanSuzuki, KenichiroHirai, YoshikazuSvetovoy, Vitaly B.Hirtz, MichaelSwami, NathanHjelme, Dag RoarSyam, WahyudinHo, Megan Yi-PingSzczepanik, BeataHoath, StephenSznitman, JosueHoettges, KaiSzulczyński, BartoszHoffmann, RolfSzymborski, TomaszHomsy, AlexandraTabib-Azar, MassoodHonda, TakashiTaboryski, Rafael J.Hong, SukjoonTaboryski, RafaelHoshiba, TakashiTabrizian, RoozbehHossain, ShakhawatTadmor, RafaelHossan, Mohammad RobiulTahara, HironobuHowlader, MatiarTaheri-Tehrani, ParsaHsieh, KuangwenTakahashi, HidetoshiHsu, Wei-LunTakashima, YuzuruHu, ChunxiaoTalaat, AhmedHu, GuoqingTaler, JanHu, ZhongxuTallaire, AlexandreHua, TaoTamarin, OllivierHuang, Chi-HsienTan, Lling LlingHuang, Kuo-ChengTan, JifuHuang, Po-HsunTan, SayHuang, Chih-HsienTan, Chee-KeongHuang, Shyh-ChourTanabe, KatsuakiHuang, HuTanaka, YoHuang, QijinTang, ShiyangHuang, ShengxiTang, JinyaoHuang, YanboTang, WilkinHuang, Yu-HsiTanikawa, TomoyukiHuang, YingTanyeri, MelikhanHuck, AlexanderTao, KaiHülsenberg, DagmarTao, LeiHummon, MatthewTardin, CatherineHunstig, MatthiasTasco, VittoriannaHuo, DehongTassieri, ManlioHwang, YongsungTatar, ErdincHwang, David J.Tavacoli, Joe W.Hwang, Suk-WonTemel, Fatma ZeynepHwang, Dong-HwanTemiz, YukselHwang, YonghaTesta, GenniHwang, Chi-HungTetienne, Jean-PhilippeHwang, Jae YounThalhammer, GregorHwu, Jenn-GwoThielemann, ChristianeHyun, Kyung-AThijssen, JosIatsunskyi, IgorThorsen, ToddIgnés-Mullol, JordiTian, YingtaoIkmo, ParkTibbetts, Katharine MooreIliescu, CiprianTibbitt, MarkIliopoulos, KonstantinosTiemann, MichaelIm, Sung GapTittonen, IlkkaIno, KosukeToan, Nguyen VanIshida, TadashiToda, MasayaIshikawa, TakujiTodorov, YankoIslam, KamrulTodorov, TchavdarIto, SoTognarelli, SeleneItoh, TamitakeTonacci, AlessandroIwami, KentaroTorres-Diaz, IsaacIwase, EijiTorrilhon, ManuelIwata, TatsuyaTortschanoff, AndreasIza, FelipeTorunbalci, Mustafa MertJackson, MarkTosello, GuidoJackson, NathanToyama, ShigeruJakieła, SławomirTrotta, GianlucaJakubik, Wiesław P.Trucco, AndreaJames, SagilTrung, Tran QuangJang, JongMoonTruong, Vi KhanhJayaram, Dhanya ThamaraparambilTrusiak, MaciejJeon, SeokwooTsai, Chia-HungJeong, YaesukTsai, Ming-Yi.Jeong, Ok ChanTsai, Ang-ChenJeong, Chang KyuTsamis, ChristosJeong, Jae-WoongTsay, Chien-YieJeong, Seung HeeTseng, Sheng-HsiangJia, RuTseng, Kuo-HsiungJia, YuTseng, Victor Farm-GuooJian, JieTseng, Wei-TsuJiang, SongTserepi, AngelikiJiang, KunTsiokos, DimitrisJiao, PengchengTsuchiya, YoshishigeJin, XiaoliangTsukamoto, TakashiroJing, WumingTullis, IainJing, QingshenTung, Chih-KuanJo, Sae ByeokTwaha, SsennogaJohnson, StephanieTzounis, LazarosJones, Matthew RUchikoba, FumioJones, PhilipUeda, TaroJones, J CliffUlyashin, Alexander G.Joshi, NiravkumarUlz, PeterJoung, DaehaUm, HandonJoung, Hyou-ArmUmasankar, YogeswaranJovanović, DraganaUrban, Matthew W.Juárez, Jaime JavierUspal, WilliamJugessur, AjuUzunlar, ErdalJung, SungjuneVAI, Mang IJung, DaewoongValli, AngeloJung, HyungilVan De Put, MaartenJung, Hyun JunVan Der Meer, Th.H.Juodkazis, SauliusVan Der Sluis, OlafJurado-Sánchez, BeatrizVan Erps, JurgenKaajakari, VilleVan Ostayen, Ron A.J.Kacem, NajibVan Roij, ReneKacher, JoshVanfleteren, JanKadasala, Naveen ReddyVara, DinaKahrs, Lueder A.Varghese Chacko, JenuKaiser, SusannaVasdekis, Andreas E.Kaji, NoritadaVazquez, Rebeca MartinezKalantar-Zadeh, KouroshVazquez, MaribelKale, AkshayVelha, PhilippeKaltsas, GrigorisVenkataraman, ShrinivasKamath, RahulVerd, JaumeKamenetska, MashaVerotti, MatteoKameoka, JunVezzoli, AndreaKanda, KensukeVidal-Álvarez, GabrielKaneko, YoshihisaVidic, JasminaKanematsu, HideyukiViefhues, MartinaKang, Yang JunVignola, Joseph F.Kang, Dong-KuVila-Comamala, JoanKang, PilgyuVilela, DianaKang, Seong JunVinayakumar, K BKang, XinViola, FabioKanik, MehmetViollet, StéphaneKannappan, RamaswamyVisaveliya, NikunjkumarKano, ShinyaVitry, PaulineKantak, ChaitanyaVolpe, GiovanniKaplan, AndreyVolz, ThomasKarakasidis, T.E.Vourdas, NikolaosKaranassios, VassiliWada, Ken-IchiKärnfelt, CamillaWaddell, EmanuelKashaninejad, NavidWalczak, RafałKaushik, AjeetWalling, JeffreyKavehei, OmidWan, AlwinKawashima, KenjiWang, LingKaya, TolgaWang, XiaoguangKazem, NavidWang, ChengKeating, AdrianWang, XiaomuKeedwell, EdwardWang, Dung-AnKehl, FlorianWang, PengtaoKeleher, Jason J.Wang, ZhisongKeller, ClémentWang, ZhenfengKelley, JoshuaWang, HaoKeszler, AgnesWang, YeqingKhalid, AtaWang, JingKhaliq, JibranWang, HaidongKhan, MuhammadWang, GangKhoshmanesh, KhashayarWang, JiangxinKiani, AmirkianooshWang, HongboKiefer, RudolfWang, GuoanKilic, Zekai MuratWang, PengKim, JaeyounWang, TaoKim, DaewonWang, QiuguKim, KyunghoonWang, WeiKim, Chang-JinWang, QiKim, Sung JaeWang, YaKim, Jin SikWang, XiaoenKim, JehyungWard, TomKim, SangkilWare, TaylorKim, Chang NyungWarkiani, Majid EbrahimiKim, KwanohWasserman, DanielKim, ChulhongWaugh, David GarrethKim, SangHyeonWei, KayaKim, Sung-JinWeiland, JamesKim, YoonseobWeinbub, JosefKim, DonginWeisensee, PatriciaKim, Hee-SeokWeng, XuanKim, Dae-YoonWhitby, Catherine PKim, JaekyunWhyte, GraemeKim, JinwookWieringa, FokkoKim, SeokminWilliams, Stuart J.Kim, Hong NamWitte, HartmutKim, JaeminWlodkowic, Donald WlodzimierzKim, Chung-SooWojtasiak, WojciechKirchner, RobertWoo, Jong-KwanKirihara, SoshuWood, Graham S.Kirillova, AlinaWood, Troy D.Kitamura, AkiraWu, XiaojianKitchen, PhilipWu, ZhiguangKityk, IwanWu, Hung-ChinKnappe, SvenjaWu, GuoqiangKnospe, Carl R.Wu, JiandongKo, HyounghoWu, KaiKo, SeungDeokWu, NanKo, Seong-YoungWu, ChengyuanKobayashi, AtsushiWu, HengKobayashi, MakikoWu, TaoKobtsev, SergeyWu, XinKoh, AhyeonWuethrich, AlainKoh, Kah HowXi, WeixianKokkoris, GeorgeXia, WenjieKoltay, PeterXiang, LiminKonda Gokuldoss, PrashanthXianyu, YunleiKondoh, JunXiao, BoqiKong, Tian FookXie, YuliangKönözsy, LászlóXie, ShangranKonstantaki, MariaXie, ZuotiKontziampasis, DimitriosXie, ZhijianKoo, ChiwanXie, SaienKootala, SujitXing, GuichuanKoppányi, ZoltánXiong, XingguoKoppes, RyanXu, WeixingKorivi, NagaXu, JiawenKorobiichuk, IgorXu, WeiheKorsunsky, Alexander M.Xu, PengKorvink, JanXu, YongKotsuchibashi, YoheiYadav, ChandanKottapalli, Ajay Giri PrakashYadavali, SagarKouh, TaejoonYagasaki, TakumaKoumoulos, Elias P.Yahiaoui, RédaKouza, MaksimYamane, DaisukeKovalchenko, Andrii M.Yan, DayunKozai, TakashiYan, JingKozlowska, JustynaYan, JiwangKraft, ThomasYang, Ya-TangKrebs, GuillaumeYang, MingKreth, PhillipYang, Jeon-WookKrolczyk, GrzegorzYang, HaoKtena, AphroditeYang, YunzeKulagin, RomanYang, Teng-ChunKulinich, SergeiYano, MitsuakiKumar, PankajYao, JeffreyKumar, AnilYap, Yiing ChiingKumar, SandeepYapici, Murat KayaKumar, AvneeshYazdi, SadeghKumar, VivekYe, HuaiyuKumavor, Patrick D.Ye, EnyiKung, Yu-ChunYeh, Li-HsienKuo, Wen-chengYeh, Min-HsinKurlyandskaya, Galina V.Yeh, Yin-TingKwok, Chee YeeYen, Shih-ChengKwon, Soon-HongYeo, Joo ChuanKwon, Kye-SiYeom, TaihoKwon, SungkyuYeom, JunghoonLa Parola, ValeriaYildirim, AdemLacava, CosimoYin, XiaodongLagarde, FlorenceYoo, ByungseokLai, Chien-ChihYoon, Hyeun JoongLai, Ying-ChihYoon, HyeonseokLai, Chin WeiYossifon, GiladLai, Wen ChengYou, ShujieLam, RaymondYu, Jae WoongLang, UdoYu, Feiqiao BrianLang, WalterYu, Ruei-SungLao, Ka UnYu, Kee-HoLauga, EricYu, Chin-pingLaurenti, MarcoYu, YunLauritano, DorinaYu, XingeLavrentovich, OlegYu, Ki JunLawrie, BenjaminYu, ZetaLayani, MichaelYu, YanhaoLazarus, NathanYuan, JunqiLe, NamTuanYuan, LeiLe Donne, AlessiaYuan, QuanLe Gac, SéverineYubero, FranciscoLe Pioufle, BrunoYue, TaoLe Traon, OlivierYurchenko, OlenaLeccese, FabioZaghari, BaharehLechuga, YolandaZagnoni, MicheleLedochowitsch, PeterZagórski, IreneuszLee, Boon GiinZahn, JeffreyLee, Tae YoonZaman, Ashraf UzLee, Jiun-hawZangari, GiovanniLee, JinkeeZareinia, KouroshLee, MinbaekZarifi, Mohammad H.Lee, Sun-KyuZayachuk, YevhenLee, JaehyunZeidler, HenningLee, Byung ChulZenati, Marco A.Lee, Seung-BeckZergioti, IoannaLee, Eon SooZhang, XianLee, Seok JaeZhang, XumingLee, Jae-UngZhang, HuananLee, VivianZhang, ZiyangLee, Sung SikZhang, XiaoLee, Seung-wooZhang, YanzhenLee, KeekeunZhang, RenyunLee, SunwooZhang, WenxuLee, Jeong HoonZhang, YunyanLee, Ying-ChiehZhang, XinquanLee, Hyunjoo JennyZhang, YuhaoLee, Hyeong JaeZhang, Xueqiang AlexLee, ChangwonZhang, JunLei, Kin FongZhang, DiLesch, AndreasZhang, RujingLevi, AlessandroZhang, HuairuoLi, HaijunZhang, NanLi, ZidaZhang, XupingLi, Chun-HsingZhang, RuiLi, YuanyuanZhang, ChaoLi, Wei-ChangZhang, XiangLi, Paul C.H.Zhang, HongtiLi, JinxingZhang, XiaoliangLi, HeZhang, ZhienLi, LinZhang, YiLi, Sheng-ShianZhang, YongLi, YangminZhao, HongLi, ZhenglongZhao, ChunLi, WendiZhao, RuogangLi, ZhengweiZhao, HangboLi, Ming-HuangZhao, YudaLi, QingZhao, YulongLi, YiyanZhao, PingLi, TaoZheng, BoLi, JiashenZheng, YuebingLi, XiangZheng, YiLi, YifanZhong, LiLiang, MengbingZhou, BingpuLiang, FengZhou, RanLiao, MeiyongZhu, HeLiao, XiaozhouZhu, LianhuaLim, Chun YeeZhu, HaoshenLim, SoomanZhu, WeiLin, LinhanZhu, YingLin, Yuan-PinZimmerman, William B.Lin, Chih-mingZolfagharian, AliLIN, YuankunZotti, LindaLin, GungunZozulya, AlexeyLin, ZuantaoZuo, LijianLin, Chih-LangZuo, LeiLin, Jr-LungŽuperl, Uroš

